# Reduction of Spike-like Noise in Clinical Practice for Thoracic Electrical Impedance Tomography Using Robust Principal Component Analysis

**DOI:** 10.3390/bioengineering12040402

**Published:** 2025-04-09

**Authors:** Meng Dai, Xiaopeng Li, Zhanqi Zhao, Lin Yang

**Affiliations:** 1 Department of Biomedical Engineering, The Fourth Military Medical University, Xi’an 710032, China; daimeng@fmmu.edu.cn; 2 Innovation Research Institution, Xijing Hospital, The Fourth Military Medical University, Xi’an 710032, China; 3 State Key Laboratory of Radio Frequency Heterogeneous Integration, Shenzhen University, Shenzhen 518060, China; 4 School of Biomedical Engineering, Guangzhou Medical University, Guangzhou 511436, China; zhanqizhao@gzhmu.edu.cn; 5 Department of Critical Care Medicine, Peking Union Medical College Hospital, Beijing 100730, China; 6 Department of Aerospace Medicine, The Fourth Military Medical University, Xi’an 710032, China

**Keywords:** electrical impedance tomography, spike-like noise, robust principal component analysis

## Abstract

Thoracic electrical impedance tomography (EIT) provides real-time, bedside imaging of pulmonary function and has demonstrated significant clinical value in guiding treatment strategies for critically ill patients. However, the practical application of EIT remains challenging due to its susceptibility to measurement disturbances, such as electrode contact problems and patient movement. These disturbances often manifest as spike-like noise that can severely degrade EIT image quality. To address this issue, we propose a robust Principal Component Analysis (RPCA)-based approach that models EIT data as the sum of a low-rank matrix and a sparse matrix. The low-rank matrix captures the underlying physiological signals, while the sparse matrix contains spike-like noise components. In simulation studies considering different spike magnitudes, widths and channels, all the image correlation coefficients between RPCA-processed images and the ground truth exceeded 0.99, and the image error of the original fEIT image with spike-like noise was much larger than that after RPCA processing. In eight patient cases, RPCA significantly improved the image quality (image error: *p* < 0.001; image correlation coefficient: *p* < 0.001) and enhanced the clinical EIT-based indexes accuracy (*p* < 0.001). Therefore, we conclude that RPCA is a promising technique for reducing spike-like noise in clinical EIT data, thereby improving data quality and potentially facilitating broader clinical application of EIT.

## 1. Introduction

Electrical impedance tomography (EIT) has emerged as a valuable tool in thoracic medicine, offering real-time, bedside imaging of pulmonary ventilation and perfusion through the analysis of chest surface impedance signal [1,2]. In critical care settings, EIT has demonstrated its versatility in various applications, including assessing lung recruitability [3,4], optimizing positive end-expiratory pressure (PEEP) [5], evaluating ventilation–perfusion matching [6], and guiding mechanical ventilation weaning strategies [7,8]. Especially during the COVID-19 pandemic, this situation further highlighted EIT’s clinical utility, as it was extensively employed to monitor and evaluate treatment efficacy in severely ill patients [9,10,11,12], yielding promising results. Beyond critical care, EIT has found applications in respiratory medicine, particularly in the assessment of obstructive lung diseases. Its ability to detect regional variations in airflow has proven useful in evaluating conditions such as asthma [13], chronic obstructive pulmonary disease (COPD) [14,15,16], and cystic fibrosis [17,18]. Recent studies have also explored EIT’s potential in pulmonary rehabilitation [19], both for critically ill patients and those with chronic respiratory conditions. These investigations have demonstrated the feasibility and efficacy of EIT-guided rehabilitation protocols.

Although EIT has certainly helped clinical decision-making in specific situations, providing valuable insights that benefit certain patients, it is crucial to acknowledge that the technique has not yet improved enough to become a routine bedside monitoring tool in daily clinical practice, despite its potential. The primary challenge is that it is mathematically an ill-posed and ill-conditioned inverse problem, making the imaging process highly sensitive to minor measurement disturbances, which can easily result in significant artifacts in the reconstructed images. But the clinical environment inherently contains various disturbances, such as patient movement, perspiration, therapeutic procedures, and routine nursing tasks, which inevitably introduce different noise into EIT measurements [20]. Thus, reducing the impact of these clinical interferences on EIT measurement data has become a critical problem to overcome for its broader clinical applications.

In fact, EIT researchers have long been aware of and attempted to address this issue, as shown in Table 1. However, the above methods did not account for the data structural characteristics of normal EIT signals and noisy signals, which is precisely the focus of this study. For most clinical applications, EIT measurement disturbances can be classified into two main categories. The first type involves electrode contact issues due to various reasons [2]. While most current commercial systems can locate poorly connected electrodes, it typically takes several minutes for medical staff to address and correct the problem (e.g., by applying saline solution or conductive gel) before normal data acquisition can resume. The second type is caused by patient movement, either active or passive. In these cases, although electrode contact remains unaffected, the overall data baseline experiences a shift, lasting for a short duration (several seconds). Regardless of type, these disturbances share common characteristics: they produce signals of large magnitude (several times the amplitude of respiratory signals) and occupy a relatively small portion of the overall EIT data volume (if EIT data are collected at 20 fps for half an hour, the total dataset contains 36,000 points; electrode contact disturbances typically affect several thousand points and movement disturbances affect several hundred points). Therefore, compared to the entire EIT dataset in clinical practices, these “bad data” can be considered as spike-like signals.

From a signal processing point of view, it is reasonable to formulate the acquired EIT dataset (before imaging) as a matrix. In this representation, the rows of the matrix correspond to the number of EIT measurement channels (typically 192 or 208 rows), while the columns represent the number of time points in the EIT measurement. Within this matrix framework, spike-like noise can be considered as disturbances affecting data at a small portion of specific time points. Given that the total volume of EIT data is often much larger than the number of data points contaminated by spike-like noise, these disturbances can be characterized as sparse relative to the entire EIT dataset. Furthermore, it is noteworthy that all EIT channels collect signals from the human thorax, resulting in a high degree of similarity among these signals. This characteristic leads to the EIT data matrix exhibiting a low-rank property.

Under the above assumption, this study proposes a spike-like noise reduction method for EIT data matrices based on Robust Principal Component Analysis (RPCA) [30,31]. RPCA is a matrix decomposition technique that separates a matrix into a sparse component and a low-rank one. Its key advantage lies in its ability to effectively extract low-dimensional structures from data, even in the presence of significant noise or outliers. Thus, we hypothesize that it is possible to effectively decompose a thoracic EIT data matrix, containing spike-like noise, into a low-rank component representing the underlying respiratory signal and a sparse component representing the noise, using RPCA. Furthermore, is it possible to improve the quality of reconstructed EIT images and the accuracy of derived clinical indices by applying RPCA to remove spike-like noise from EIT data obtained in both simulated and clinical settings?

The remainder of the paper is organized as follows: Section 2 introduces the RPCA framework and its application to EIT noise reduction, including validation studies for simulated and human datasets. Section 3 presents comparative results on noise reduction efficiency and EIT images, as well as the indices. Section 4 discusses clinical implications, limitations, and future directions. Conclusions are summarized in Section 5.

## 2. Materials and Methods

### 2.1. Formulation Using RPCA

In our study, we first examine the low-rank property of EIT data matrices, noting that the sparsity of spike-like noise is inherently satisfied.

#### 2.1.1. The Low-Rank Property of an EIT Data Matrix

**Figure 1:**
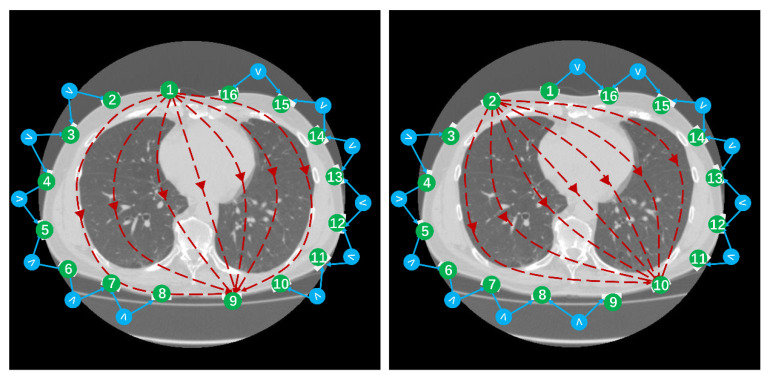
The measurement principle of EIT for the opposite excitation method, where one data frame contains 192 boundary voltage values.

The measurement principle of EIT is shown in Figure 1 below: a chest CT slice and corresponding EIT electrode positions, where green circles represent electrode positions. As an example, when current is input from electrode 1 and output from electrode 9, the other electrodes measure the boundary voltage signals, but electrodes 1 and 9 do not participate in the measurement. Thus, under the same excitation electrode pair, 12 measured voltage values are formed. Then, the excitation electrode pair is switched, such as switching to electrode 2 for input current and electrode 10 for output current. The other electrodes similarly measure the boundary voltage signals, generating another 12 measured voltage values. This process continues, with the excitation electrode pair rotating a full circle through 16 switches, thus obtaining 12 × 16 = 192 boundary voltage values.

EIT data matrix consists of EIT measurement vectors that correspond to the boundary voltages at different time points. For example, using the opposite current excitation–adjacent voltage measurement mode for a 16-electrode system, one EIT measurement vector at the moment of t can be denoted as $v_{t}=[v_{1},v_{2},v_{3},v_{4},\ldots ,v_{n},\ldots ,v_{192}]\in {\mathbb{R}}^{192\times 1}$ and $v_{n}$ is the voltage at the nth channel. Further, the EIT data matrix resulting from measurement within the T time period can be organized as

$$V_{T}=[v_{1},v_{2},v_{3},v_{4},\ldots ,v_{t},\ldots ,v_{T}]\in R^{192\times T}$$

To the low-rank property of EIT data matrix, one EIT data matrix was obtained by performing measurement within 2368/20 = 118.4 s (2368 and 20 is the total frame number and frame rate of data acquisition) in a healthy male using a commercial EIT system (VenTom-100, MediasMed, Suzhou, China). Then, singular value decomposition was implemented for the EIT data matrix.

(1) $$V_{T}=U\Sigma Q^{T}$$

where U and Q are the left and right singular vectors, Σ is the singular values matrix with non-negative real numbers on the diagonal.

**Figure 2:**
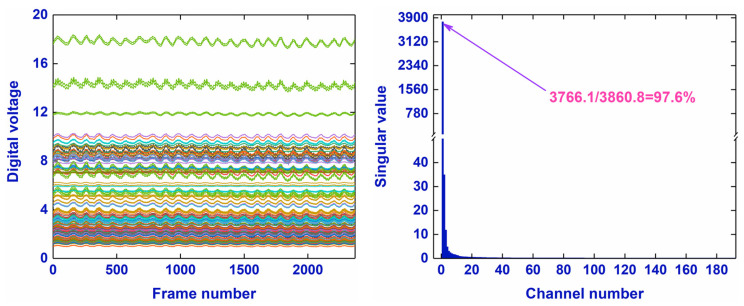
The respiratory wave calculated from the sum of digital voltages at all channels of one EIT data matrix from measurements within 2368/20 = 118.4 s (2368 and 20 is the total frame number and frame rate of data acquisition) in a healthy male using a commercial EIT system (VenTom-100, Medias, Suzhou, China), and the singular values of the EIT data matrix.

The first singular value accounts for the vast majority (97.6%) of the total singular value sum, suggesting the approximately low-rank property of the EIT data matrix (Figure 2).

#### 2.1.2. Principle of RPCA

RPCA is an advanced version of traditional Principal Component Analysis (PCA) designed to manage real-world datasets that may contain outliers or spike-like noise [32]. RPCA decomposes the observed data matrix into the sum of two parts: a low-rank matrix that captures the main structure of the data and a sparse matrix that accounts for outliers or noise. By efficiently isolating and eliminating these outliers while retaining crucial information, RPCA is widely used in fields like signal processing.

Specifically, given an observed data matrix $D\in {\mathbb{R}}^{m\times n}$, RPCA seeks matrices L and S such that:

(2) D=L+S

where L captures the underlying structure of the data and S represents the outliers and corruptions. To recover L and S, the following optimization problem needs to be solved:

(3) $$\underset{L,S}{\min}\;\;{\left\|L\right\|}_{*}+\lambda {\left\|S\right\|}_{1},\;\;\;\;subject\;to\;\;D=L+S$$

where ${\left\|L\right\|}_{*}$ denotes the nuclear norm (for a matrix $L\in {\mathbb{R}}^{m\times n}$, ${\left\|L\right\|}_{*}=\sum_{i=1}^{\min\left(m,n\right)}\sigma_{i}\left(L\right)$ where $\sigma_{i}\left(L\right)$ represents the *i*-th singular value of the matrix L) of L promoting low rank, ${\left\|S\right\|}_{1}$ is the $\ell_{1}\text{-}\mathrm{norm}$ (sum of the absolute value of all entries) of S promoting sparsity, and λ denotes a positive regularization parameter balancing the two terms. In general, a large λ results in a small rank of S. In EIT, $D\in {\mathbb{R}}^{m\times n}$ represents the measured data with m channels and n points, L is the measured data without spike-like noise, and S means spike-like noise.

In this study, we solve problems (2) using the Alternating Direction Method of Multipliers (ADMM), resulting in

(4) $$L\left(L,S,\mu \right)=\;{\left\|L\right\|}_{*}+\lambda {\left\|S\right\|}_{1}+\langle \mu ,D-L-S\rangle +\frac{\lambda_{0}}{2}{\left\|D-L-S\right\|}_{F}^{2}$$

where λ is a positive scalar, μ is the Lagrange multiplier. By minimizing the $L\left(L,S,\mu \right)$, we can finally obtain L (the measured EIT data without spike-like noise) and S (spike-like noise). The Frobenius norm ${\left\|\cdot \right\|}_{F}$ means that, for a matrix $X\in {\mathbb{R}}^{m\times n}$, ${\left\|X\right\|}_{F}=\sqrt{trace\left(X^{T}X\right)}$.

The update for L is given by:

(5) $$L_{k+1}=\arg\underset{L}{\min}\;{\left\|L\right\|}_{*}\;+\frac{\lambda_{0}}{2}{\left\|D-L-S_{k}+\frac{\mu_{k}}{\lambda_{0}}\right\|}_{F}^{2}$$

using Singular Value Thresholding (SVT), we solve it as follows:

(6) $$\begin{array}{l} D-S_{k}+\frac{\mu_{k}}{\lambda_{0}}=U\Sigma V^{T}\;\;\\ \tilde{\Sigma }=\max(\Sigma -\frac{1}{\lambda_{0}},0)\;\\ L_{k+1}=U\tilde{\Sigma }V^{T} \end{array}$$

where $\lambda_{0}$ is also a positive scalar as a regularization parameter and $U\Sigma V^{T}$ means the singular value decomposition to $L_{k}$. ∑ represents the diagonal elements of the diagonal matrix Σ.

The update of S is given by:

(7) $$S_{k+1}=\arg\underset{S}{\min}\lambda {\left\|S\right\|}_{1}+\frac{\lambda_{0}}{2}{\left\|D-L_{k+1}-S+\frac{\mu_{k}}{\lambda_{0}}\right\|}_{F}^{2}$$

using element-wise soft thresholding, we solve it as follows:

(8) $$\begin{array}{l} X=D-L_{k+1}+\frac{\mu_{k}}{\lambda_{0}}\\ S_{k+1}=\mathrm{sign}(X)\cdot \max(|X|-\frac{\lambda }{\lambda_{0}},0) \end{array}$$

Update the Lagrange multiplier μ

(9) $$\mu_{k+1}=\mu_{k}+\lambda_{0}(D-L_{k+1}-S_{k+1})$$

Based on Lemma 3.1 in [33], the pair $\left(L_{k},S_{k}\right)$ converges to the unique optimal solution to problem (2). The specific calculation (Algorithm 1) is outlined below.
**Algorithm 1** Robust Principal Component Analysis

1: Initialization: 
$S_{1}=0$, λ=0.01, $\lambda_{0}=10$, $\mu_{1}=0$, $\epsilon =10^{-8}$

2: **while** $\|D-L-S{\|}_{F}>\epsilon$ **do**

3:      Solve $L_{k+1}=\arg\min\;L(L_{k},S_{k},\mu_{k})$

4:      Solve $S_{k+1}=\arg\min\;L(L_{k+1},S_{k},\mu_{k})$

5:      $\mu_{k+1}=\mu_{k}+\lambda_{0}(D-L_{k+1}-S_{k+1})$

6: **end while**

7: **Output:** $L_{k}$
$,S_{k}$


### 2.2. Numerical Simulations

#### 2.2.1. Generation of Respiratory EIT Boundary Voltages

**Figure 3:**
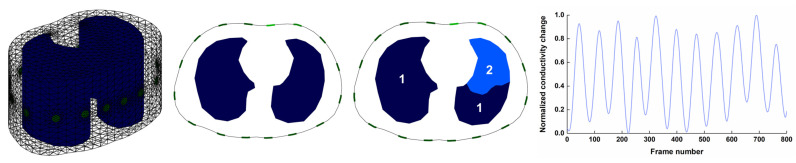
Generation of respiratory EIT boundary voltages: 2.5D finite element model and normalized conductivity change with time. The conductivity of Area 1# was set to the normalized conductivity change, whereas the conductivity in Area 2# was set to 0.15 times the normalized values.

A 2.5D finite element model, created in the EIDORS 10.0 platform [34], was composed of 21,396 triangular elements and included the realistic thorax and lung contours segmented from a CT image located between the fourth and fifth ribs of an adult male, as shown in Figure 3. In this finite element model, 16 disk electrodes with a 1 cm diameter were equidistantly spaced on the surface of the thorax. To simulate the breathing process, the reciprocal of the sum of boundary voltages in all measurement channels recorded from a healthy male (age: 36 years; weight: 75 kg; height: 175 cm) for about 40 s was normalized to set the conductivity of two lung areas, as shown in Figure 3. The conductivity of the non-lung area was set to 1 S/m. The conductivity of Area 1# was set to the normalized conductivity change, whereas the conductivity in Area 2# was set to 0.15 times the normalized values. Further, by applying a 1 mA current for excitation, the time-series EIT boundary voltages were obtained with the use of the opposite current excitation–adjacent voltage measurement mode, respectively.

#### 2.2.2. Spike-like Noise Synthesis and Imaging

The performance of RPCA on resisting spike-like noise was explored in three aspects: spike magnitude, spike width, and spike channels in EIT measurement.

In terms of spike magnitude, the 35th channel of the 299th frame was randomly selected, and then its boundary voltage was multiplied by 1, 2, 3, 4, 5, 6, 7, 8, 9, 10, 20, 30, 40, 50, 60, 70, 80, 90, and 100 (19 types in total), respectively. The boundary voltage in the 255th frame, which was the inspiration start time of the breathing cycle nearest to the 299th frame, was used as the reference to reconstruct time-series EIT images. The reconstructed EIT image without spike-like noise in the 299th frame was deemed as the ground truth, and all reconstructed EIT images from RPCA-processed boundary voltages with spike-like noise in the 299th frame were compared to the ground truth.

In terms of spike width, the time-series boundary voltages in the 35th channel with 11 kinds of consecutive point widths (point width: 5, 10, 20, 30, 40, 50, 60, 70, 80, 90, and 100) were multiplied by 50. The boundary voltage in the 255th frame was also used as the reference to reconstruct time-series EIT images. For each spike width, the functional EIT (fEIT) image (standard deviation method) calculated from time-series EIT images without spike-like noise was considered as the ground truth. Then, the fEIT image of time-series EIT images reconstructed from RPCA-processed boundary voltages with spike-like noise was compared to the ground truth.

In terms of spike channel, the time-series boundary voltages from 300th to the 350th frame in 14 different channel numbers (start channel: 1st; channel numbers: 5, 10, 20, 30, 40, 50, 60, 70, 80, 90,100, 150, 190, and 192) were multiplied by 50. The boundary voltage in the 255th frame was also used as the reference to reconstruct time-series EIT images. For each spike channel, the functional EIT (fEIT) image (standard deviation method) calculated from time-series EIT images without spike-like noise was considered as the ground truth. Then, the fEIT image of time-series EIT images reconstructed from RPCA-processed boundary voltages with spike-like noise was compared to the ground truth. In this study, the GREIT algorithm was used to reconstruct EIT images.

Additionally, to explore the ability of RPCA to resist spike noise when the lung conductivity was heterogeneously distributed, the left lung area was divided into two parts with different conductivities. The conductivity of Area 1 was set to the normalized boundary voltages, whereas the conductivity of Area 2 was set to 0.15 times the normalized boundary voltages. The spike width and spike magnitude were randomly selected, and they were 61 and 27. Also, the spike channels (50 in total) were randomly selected. The boundary voltages with spike noise were processed by a low-pass filter (order: 3, cutoff frequency: 60 bpm), a median filter (order: 20), and RPCA, respectively. Then, the fEIT image of time-series EIT images reconstructed from boundary voltages processed by each method was compared to the ground truth.

### 2.3. Human Experiments

Eight patients were involved in this study. The time-series boundary voltages with spike noise were selected. For example, the first patient suffered from a left pulmonary nodule and was scheduled for laparoscopic lung parenchymal resection in the Department of Thoracic Surgery of Tangdu Hospital, Air Force Medical University, Xi’an, China. EIT was used to evaluate the effect of laparoscopic lung parenchymal resection. Before anesthesia, thoracic EIT data were continuously collected for about 10 min in the supine position by using the commercial EIT system (VenTom-100, MidasMed, Suzhou, China). During data collection, the patient kept spontaneous breathing, but his deliberate movement, medical treatment, and nursing were not restricted.

To estimate the fEIT image of the respiratory cycle corrupted by spike noise as accurately as possible, the adjacent breathing cycle without spike noise was selected, and its time-series EIT images were used to calculate the reference fEIT image.

All human trials have been approved by the Human Research Ethics Committee of Tangdu Hospital of the Fourth Medical University (NO. K202212-13), the Ethics Committees of the Fourth Medical University (KY20224101-1). Written informed consent has been obtained from all patients.

### 2.4. Image Analysis

To quantify the performance of RPCA in resisting spike-like noise, two image metrics and four clinical EIT-based indexes were employed.

Two image metrics include the image correlation coefficient ${\mathrm{Img}}_{corr}$ and image error ${\mathrm{Img}}_{err}$, which evaluate the similarity between the original (or reference) EIT image without spike ${\mathrm{EIT}}^{original/ref}$ and the RPCA-processed EIT image with spike-like noise ${\mathrm{EIT}}^{RPCA}$.

(10) $${\mathrm{Img}}_{corr}=\frac{\sum_{m=1}^{32}\sum_{n=1}^{32}\left({\mathrm{EIT}}_{m,n}^{RPCA}-\bar{{\mathrm{EIT}}^{RPCA}}\right)\left({\mathrm{EIT}}_{m,n}^{original/ref}-\bar{{\mathrm{EIT}}^{original/ref}}\right)}{\sqrt{\left(\sum_{m=1}^{32}\sum_{n=1}^{32}{\left({\mathrm{EIT}}_{m,n}^{RPCA}-\bar{{\mathrm{EIT}}^{RPCA}}\right)}^{2}\right)\left(\sum_{m=1}^{32}\sum_{n=1}^{32}{\left({\mathrm{EIT}}_{m,n}^{original/ref}-\bar{{\mathrm{EIT}}^{original/ref}}\right)}^{2}\right)}}$$

where $\bar{{\mathrm{EIT}}^{RPCA}}$ and $\bar{{\mathrm{EIT}}^{original/ref}}$ are the average values of all pixels in ${\mathrm{EIT}}^{RPCA}$ and ${\mathrm{EIT}}^{original/ref}$.

The ratio of the RMS error of the method to its average absolute value was used to measure the deviation between the RPCA-processed image and the original/reference image.

(11) $${\mathrm{Img}}_{err}=\frac{\sum_{i=1}^{N}{\left({\mathrm{EIT}}_{i}^{RPCA}-{\mathrm{EIT}}_{i}^{original/ref}\right)}^{2}}{\sum_{i=1}^{N}abs\left({\mathrm{EIT}}_{i}^{original/ref}\right)}$$

where N denote the number of EIT images; in this study, EIT image was shown as 32 × 32 pixels and, thus, N=1024.

Four clinical EIT-based indexes involve the center of ventilation (*CoV*), global inhomogeneity (*GI*), ventral to dorsal side ratio (*VtoD*), and right to left lung ratio (*RtoL*). *CoV* quantifies the ventilation distribution influenced by gravity or lung diseases.

(12) $$CoV=\frac{\sum_{i=1}^{N}\left(l_{i}\times {\mathrm{EIT}}_{i}\right)}{\sum_{i=1}^{N}{\mathrm{EIT}}_{i}\times 100\%}$$

where $l_{i}$ is the distance of ith pixel from the ventral side.

*GI* quantifies the heterogeneity of ventilation.

(13) $$GI=\frac{\sum_{i=1}^{N}abs\left({\mathrm{EIT}}_{i}-median(\mathrm{EIT})\right)}{\sum_{i=1}^{N}{\mathrm{EIT}}_{i}}$$

*VtoD* and *RtoL* describe the regional ventilation distribution, and they were computed as the ratio of the sum of pixel values within the ventral area to that within the dorsal area, as well as right side to left side, i.e., $VtoD=\frac{\sum_{i\in ventral}{\mathrm{EIT}}_{i}}{\sum_{i\in dorsal}{\mathrm{EIT}}_{i}}$ and $RtoL=\frac{\sum_{i\in right}{\mathrm{EIT}}_{i}}{\sum_{i\in left}{\mathrm{EIT}}_{i}}$.

Further, the difference for four clinical indexes between the original/reference EIT image without spike-like noise and the RPCA-processed EIT image without spike-like noise was calculated, i.e., $CoV_{err}$, $GI_{err}$, $VtoD_{err}$ and $RtoL_{err}$. For instance, $CoV_{err}=CoV^{RPCA}-CoV^{ref}$. The independent samples *t*-test was used to compare image metrics and clinical EIT-based indexes before and after RPCA processing.

## 3. Results

### 3.1. Results of Numerical Simulations

**Figure 4:**
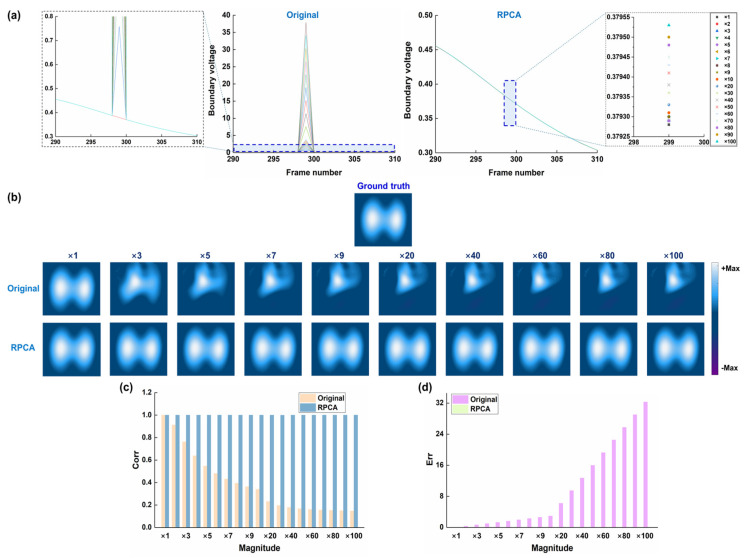
Results of the performance of RPCA in resisting different magnitudes of spike-like noise: (**a**) time-series boundary voltages with different magnitudes of spike-like noise, which was added at the 35th channel in the 299th frame, and the RPCA-processed voltages; (**b**) ground truth, which refers to the reconstructed EIT image with the boundary voltages without spike-like noise, original EIT images with different magnitudes of spike-like noise, and RPCA-processed EIT images; (**c**) image correlation coefficients between original/RPCA-processed images and the ground truth; (**d**) image error between original/RPCA-processed images and the ground truth.

Figure 4 shows the results of the performance of RPCA in resisting different magnitudes of spike-like noise. With the increasing magnitude of spike-like noise, the artifacts gradually distorted the ventilation areas. When the magnitude of spike-like noise increased to three times of boundary voltage, the ventilation areas were fully covered by the artifacts. After RPCA was processed, the ventilation areas were restored. The image correlation coefficient remained above 0.99 after RPCA was applied for all magnitudes of spike noise, but gradually decreased with increasing magnitudes of spike noise for original EIT images (before RPCA was applied). Conversely, image error was below 0.0125 after RPCA processing for all magnitudes of spike noise, while it gradually increased from 0.125 to 32.289 with increasing magnitude of spike noise. These results suggest that RPCA could deal with various magnitudes of spike noise, which range from 1 to 100 times the normal boundary voltage.

**Figure 5:**
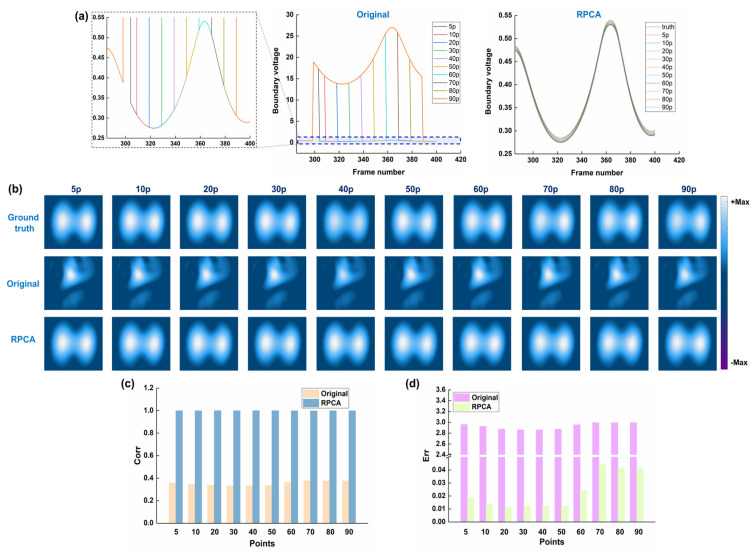
Results of the performance of RPCA in resisting different widths of spike-like noise: (**a**) time-series boundary voltages with different widths of spike-like noise, which were added at the 35th channel in the 299th frame, and the RPCA-processed voltages; (**b**) ground truth, which refers to the reconstructed EIT image before the spike-like noise was added to the boundary voltages, original EIT images with different widths of spike-like noise, and RPCA-processed EIT images; (**c**) image correlation coefficients between original/RPCA-processed images and the ground truth; (**d**) image error between original/RPCA-processed images and the ground truth.

Figure 5 shows the results of the performance of RPCA in resisting different widths of spike-like noise. For all spike-like noise widths from 5 to 90 consecutive points, the artifacts caused by noise completely obscured both sides of the ventilation areas; however, after RPCA processing, the ventilation regions were clearly observed. The image correlation coefficient was less than 0.35 for original fEIT images corresponding to all widths of spike-like noise; however, after RPCA processing, it became larger than 0.99 for all cases. The image error of the original fEIT image for each width of spike-like noise was much larger than that after RPCA processing. These results suggest that RPCA can effectively resist different widths of spike-like noise, which range from 1 to 100 points (almost one breathing cycle).

**Figure 6:**
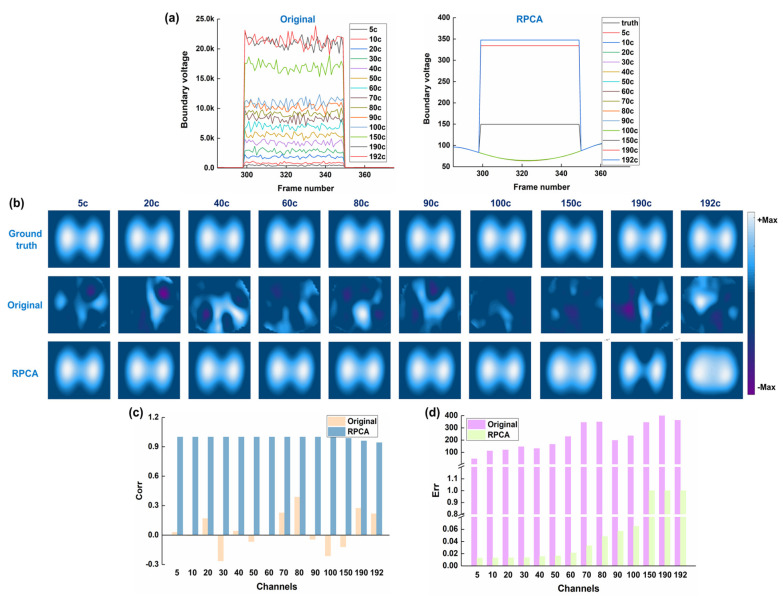
Results of the performance of RPCA in resisting different channels of spike-like noise: (**a**) time-series boundary voltages with different channels of spike-like noise, which were added at the 35th channel in the 299th frame, and the RPCA-processed voltages; (**b**) ground truth, which refers to the reconstructed EIT image before the spike-like noise was added to the boundary voltages, original EIT images with different channels of spike-like noise, and RPCA-processed EIT images; (**c**) image correlation coefficients between original/RPCA-processed images and the ground truth; (**d**) image error between original/RPCA-processed images and the ground truth.

Figure 6 shows the results of the performance of RPCA in resisting different channels of spike-like noise, ranging from 5 to 192 channels. For all cases, the ventilation regions were fully distorted in the original fEIT images, while the ventilation areas could be observed after RPCA processing. When the corrupt channels were less than 150, image correlation coefficients were all larger than 0.99, and all image errors were smaller than 0.065. However, when the corrupted channels increased to 150, image correlation coefficients decreased to 0.98, and the image error obviously increased (up to 1), suggesting that image quality has been severely degraded. These results indicate that RPCA can successfully deal with the corrupted channels below 100.

**Figure 7:**
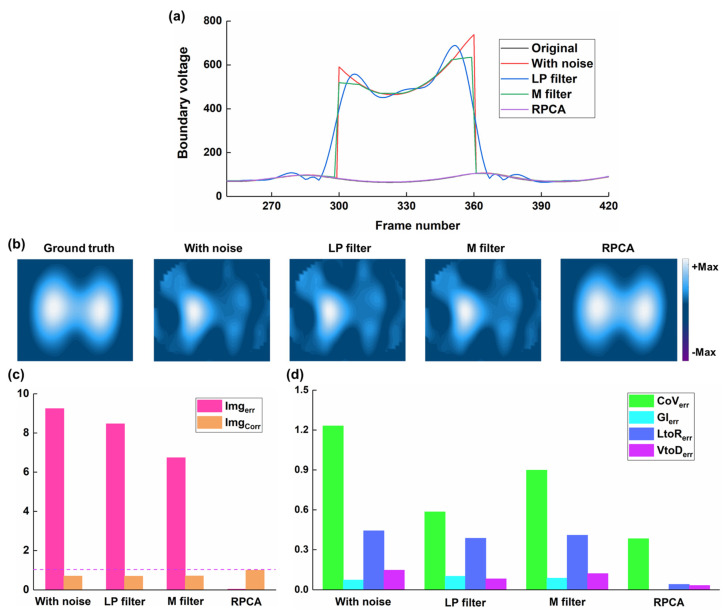
Comparison of the performance of low-pass (LP) filter, median (M) filter, and RPCA in resisting spike-like noise: (**a**) time-series boundary voltages with spike noise and results processed by low-pass filter, median filter, and RPCA; (**b**) ground truth, which refers to the reconstructed EIT image before the spike-like noise was added to the boundary voltages, the functional EIT (fEIT) images; (**c**) image quality (dotted line represent 1), and (**d**) clinical EIT-based indexes of fEIT images.

Figure 7 shows the comparison of the performance of the low-pass filter, median filter, and RPCA in resisting spike-like noise. The low-pass filter and median filter could not resist the spike noise in the boundary voltages, and thus, there were still significant artifacts in the fEIT images. But after RPCA was processed, the spike noise in the boundary voltages was removed, and the ventilation areas were restored. The image error for the fEIT image after RPCA processing was 0.0425, which is smaller than those after the low-pass filter and median filter processing (8.47 and 6.738). Also, the image correlation coefficient for the fEIT image after RPCA processing was 0.9982, whereas those after low-pass filter and median filter processing were 0.7036 and 0.7162. Moreover, significant error reduction in clinical EIT-based indices was observed in fEIT images after RPCA processing, but not with the low-pass or median filter approaches.

### 3.2. Results of Human Experiments

**Figure 8:**
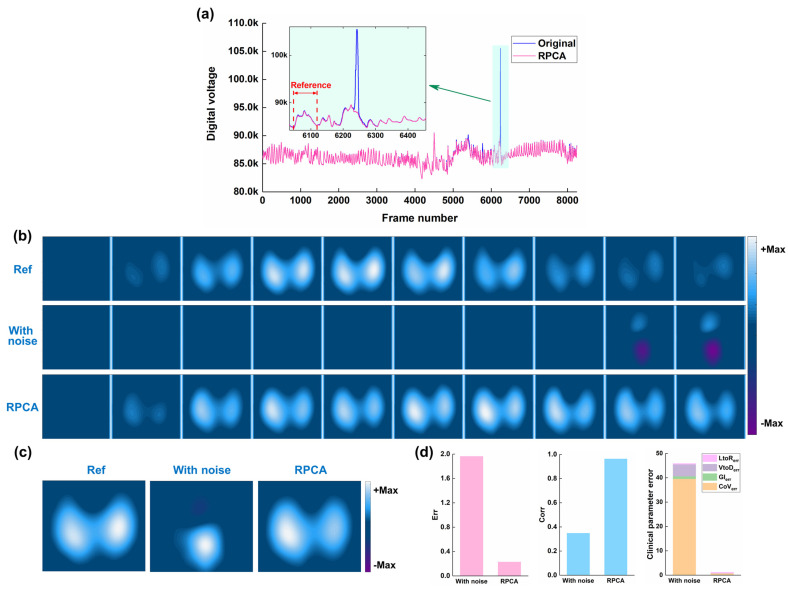
Results of the performance of RPCA in resisting spike-like noise of thoracic EIT data for a patient suffering from a left pulmonary nodule: (**a**) time-series boundary voltages, which were calculated as the sum of voltages in all channels; (**b**) time-series EIT images of reference EIT data without spike-like noise, EIT data with spike-like noise, and EIT data after RPCA processing; (**c**) functional EIT (fEIT) images; (**d**) image quality and clinical EIT-based indexes of fEIT images.

Figure 8 shows the typical results of the performance of RPCA in resisting spike-like noise of thoracic EIT data for a patient suffering from a left pulmonary nodule. As can be seen in Figure 8a, there exists an obvious spike-like noise in the thoracic EIT data, which was much larger than the normal voltage. The spike-like noise caused significant artifacts, making it difficult to identify the ventilation areas (Figure 8b). After RPCA was applied, the spike-like noise was effectively removed, and both sides of the ventilation areas were clearly observed (Figure 8b). Intuitively, the fEIT images also exhibited that the RPCA resisted the spike-like noise (Figure 8c). Compared with the reference fEIT image, RPCA reduces image error from 1.96 to 0.22 and increases the image correlation coefficient from 0.37 to 0.96 (Figure 8d). Also, RPCA greatly decreased the four clinical index errors (*CoV*err: from 39.58 to 0.75; *GI*err: from 1.05 to 0.008; *VtoD*err: from 4.91 to 0.04; *LtoR*err: from 0.36 to 0.31).

**Figure 9:**
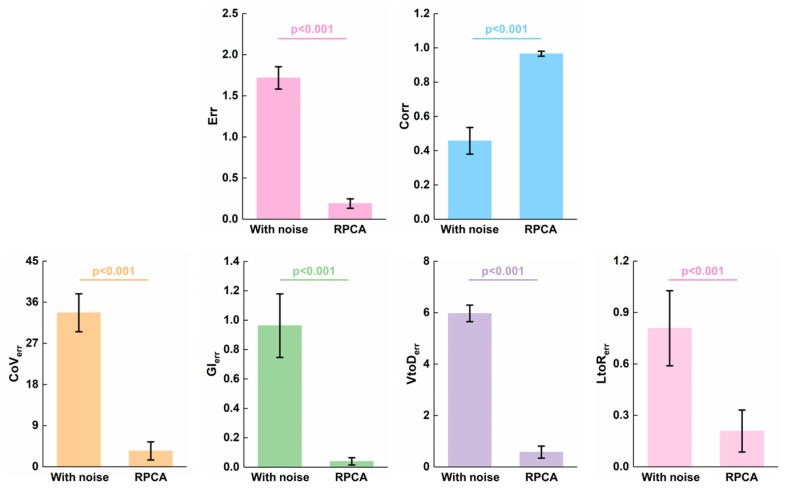
Results of the performance of RPCA in resisting spike-like noise of thoracic EIT data for eight patients: image error, image correlation coefficient, *CoV* error, *GI* error, *VtoD* error, and *LtoD* error.

Figure 9 shows the results of the performance of RPCA in resisting spike-like noise of thoracic EIT data for eight patients. As can be seen, RPCA significantly improved the image quality (image error: *p* < 0.001; image correlation coefficient: *p* < 0.001) and enhanced the clinical EIT-based indexes accuracy (*CoV* error: *p* < 0.001; *GI* error: *p* < 0.001; *VtoD* error: *p* < 0.001; *LtoD* error: *p* < 0.001).

## 4. Discussion

In this study, we initially employed the RPCA method to minimize spike-like noise in EIT signals in clinical settings. The results from our simulations demonstrated that RPCA is effective in reducing spike-like noise across a range of amplitudes and durations. We also tested the RPCA denoising method on real clinical data. While we had only worked with two typical cases so far, our early findings suggest that RPCA can significantly reduce spike-like noise and restore underlying respiratory signals, as well as EIT images. In the future, we plan to further test the method’s ability to generalize and its robustness with a larger set of clinical data.

The stability of EIT signals has long posed a significant challenge for its broader application in clinical environments. While several studies have attempted to address this issue, the EIT acquisition process in clinical settings meets various complex challenges. Achieving the level of stability seen in ECG signals is a difficult task. There are two key reasons: First, the difference in frequencies. ECG signals function at very low frequencies, allowing operational amplifiers to achieve exceptionally high input impedance in this range, which helps to counteract the impact of variations in the electrode–skin contact impedance. On the other hand, EIT excitation signals operate at much higher frequencies, usually in the tens of kilohertz range. At these higher frequencies, current analog circuit technologies are unable to implement operational amplifiers with sufficiently high input impedance, making EIT measurement circuits more sensitive to changes in contact impedance. Second, compared to ECG circuits, which only need to measure the potential at the electrodes, EIT circuits need to apply an excitation current to the measurement target. Due to the higher frequency range of EIT excitation signals, existing analog circuit technologies are unable to produce current sources with sufficiently high output impedance. Consequently, the excitation current itself fluctuates with changes in contact impedance, making it harder for subsequent circuits to control these variations. Thus, when there are motion-induced changes in the electrode–skin contact impedance, EIT signals will generate much stronger interference signals compared to ECG signals.

Also, in practice, stabilizing electrodes or patients and re-acquiring EIT signals is often not feasible. For example, when saline is injected to observe the ratio distribution of ventilation/perfusion, any signal instability can prevent the acquisition of accurate EIT images. Moreover, re-injecting hypertonic saline is unsafe. Likewise, many elderly COPD patients are unable to meet FVC standards during routine lung function and EIT tests. Asking them to exhale again can exacerbate their breathlessness, forcing doctors to halt the test for safety reasons. Consequently, improving signal stability and robustness is essential for advancing EIT in clinical settings, particularly for monitoring patients’ conditions.

This paper focuses on addressing the common spike-like noise in clinical settings by utilizing RPCA-based matrix recovery techniques. At present, our method is designed only for offline processing and analysis of EIT data. We explored several online filtering techniques, such as standard low-pass filters (e.g., Butterworth and Chebyshev 1,2, etc.), median filters for pulse signals, online PCA decomposition, discrete wavelet decomposition, and empirical mode decomposition, etc. However, these approaches were not very effective. The broad frequency spectrum of spike-like noise makes it challenging to determine an appropriate cutoff frequency for low-pass filters. While median filters work well for small pulse noise, they fail to suppress wider and larger spike-like noise and introduce additional signal delay. The other decomposition methods also proved inadequate since nearly every decomposed signal component still contained spike-like noise, making it difficult to determine which components to discard.

RPCA, on the other hand, accounts for both the low-rank nature of normal EIT signals and the sparsity of spike-like noise, resulting in superior decomposition outcomes. This suggests that broader matrix recovery techniques could have great potential for solving similar EIT signal problems. However, RPCA also faces some challenges, such as the need to empirically adjust regularization parameters for each dataset. In the future, we plan to explore techniques like the L-method and cross-validation to automate the determination of these parameters.

Additionally, it is important to note that, generally, when a particular electrode has poor contact, the measurement signals associated with that electrode become very large, approaching the saturation level of the amplifier. Conversely, when using that electrode as the excitation source, no current is injected, resulting in very small measurement values at the other electrodes. To address this issue and effectively use RPCA, for electrodes identified by the EIT system as having poor contact, we artificially set their measurement values during excitation to the amplifier’s saturation level. This approach ensures the low-rank property of the EIT measurement data.

There are several limitations in this study: (1) EIT signals contain more than just spike-like noise. During clinical measurements, we sometimes observed that respiratory rhythm signals would jump from one baseline to another, a phenomenon known as the so-called step-like noise. This often occurs due to patient positioning changes or electrode movement. We have not yet investigated whether RPCA can effectively handle this type of interference. In theory, applying first-order differentiation to step-like noise could convert it into spike-like noise, which we plan to explore further. (2) Since patients involved in this study maintained spontaneous breathing during data collection, respiratory conditions naturally varied between breathing cycles. To estimate the fEIT image of the respiratory cycle corrupted by spike noise as accurately as possible, the adjacent breathing cycle without spike noise was selected, and its time-series EIT images were used to calculate the reference fEIT image. Despite this, it is impossible to ensure complete consistency in respiratory states between the two breathing cycles. As a result, differences between the reference image and the RPCA-restored image were observed. However, results of two image quality metrics and four clinical EIT-based indexes showed that RPCA greatly improved the image quality and significantly enhanced the accuracy of the EIT-based indexes. In clinical practice, medical staff usually rely on the EIT-based indexes to objectively assess the patient’s lung ventilation. Therefore, RPCA has potential in resisting spike noise. (3) In this study, we need to tune the parameters of the RPCA algorithm to achieve better denoising effects. In the future, we will further explore adaptive parameter tuning methods to reduce manual processing. Future research will also involve testing RPCA on larger clinical datasets to assess its generalizability.

## 5. Conclusions

In this study, we first demonstrate that the RPCA technique can effectively reduce spike-like noise in EIT measurement signals, even recovering correct EIT images from a faulty one. Specifically,

- RPCA can effectively decompose a thoracic EIT data matrix, containing spike-like noise, into a low-rank component representing the underlying respiratory signal and a sparse component representing the noise
- Applying RPCA to remove spike-like noise from EIT data can improve the quality of reconstructed EIT images and the accuracy of derived clinical indices, in both simulated and clinical settings.

Although we obtained a stable respiratory EIT signal via RPCA regarding spike-like noise, the other common EIT noise, such as step-like noise, was not investigated in this study. In the future, we will explore this and develop online RPCA processing for convenience in clinical practices.

## Figures and Tables

**Table 1:** The current studies on reducing the noise of EIT data.

Author(s)	Pros	Cons
Mamatjan et al. [21]	Proposed a quantitative metric for assessing EIT data quality in real-time.	Did not offer methods for correcting or improving low-quality data.
Asfaw and Adler [22]	Proposed an automated method to detect erroneous electrodes by comparing measured data to estimates.	Sensitivity of the algorithm decreases significantly when the number of interfered electrodes is greater than three.
Adler [23]	Modeled erroneous EIT data as having infinite noise in the regularized inverse to improve EIT imaging.	The same as above
Hartinger et al. [24]	Presented an approach for real-time management of faulty electrodes based on voltage–current reciprocity, allowing image reconstruction without prior knowledge of faulty electrodes and automatic detection.	The same as above
Zhang et al. [25]	Presented an algorithm based on curve fitting to detect and filter out faulty electrode data.	Performance deteriorates when the actual number of faulty electrodes exceeds the assumed number.
Shi yanyan et al. [26,27,28,29]	Showed promise in phantom experiments for EIT data denoising for deep learning.	Clinical effectiveness remains to be further validated, as well as the challenge in obtaining large quantities of diverse clinical noise data for model training.

## Data Availability

Data are available upon reasonable request to the corresponding author.
